# scSorterDL: a deep neural network-enhanced ensemble LDAs for single cell classifications

**DOI:** 10.1093/bib/bbaf446

**Published:** 2025-09-01

**Authors:** Kailun Bai, Belaid Moa, Xiaojian Shao, Xuekui Zhang

**Affiliations:** Department of Mathematics and Statistics, University of Victoria, Victoria, BC V8P 5C2, Canada; Digital Research Alliance of Canada, Victoria, BC V8P 5C2, Canada; Digital Technologies Research Centre, National Research Council Canada, Ottawa, ON K1A 0R6, Canada; Ottawa Institute of Systems Biology, Department of Biochemistry, Microbiology, and Immunology, University of Ottawa, Ottawa, ON K1H8M5, Canada; Department of Mathematics and Statistics, University of Victoria, Victoria, BC V8P 5C2, Canada

**Keywords:** single-cell RNA sequencing, cell type annotation, penalized linear discriminant analysis, deep neural networks, swarm learning

## Abstract

The emergence of single-cell RNA sequencing (scRNA-seq) technology has transformed our understanding of cellular diversity, yet it presents notable challenges for cell type annotation due to data’s high dimensionality and sparsity. To tackle these issues, we present scSorterDL, an innovative approach that combines penalized Linear Discriminant Analysis (pLDA), swarm learning, and deep neural networks (DNNs) to improve cell type classification. In scSorterDL, we generate numerous random subsets of the data and apply pLDA models to each subset to capture varied data aspects. The model outputs are then consolidated using a DNN that identifies complex relationships among the pLDA scores, enhancing classification accuracy by considering interactions that simpler methods might overlook. Utilizing GPU computing for both swarm learning and deep learning, scSorterDL adeptly manages large datasets and high-dimensional gene expression data. We tested scSorterDL on 13 real scRNA-seq datasets from diverse species, tissues, and platforms, as well as on 20 pairs of cross-platform datasets. Our method surpassed nine current cell annotation tools in both accuracy and robustness, indicating exceptional performance in both cross-validation and cross-platform contexts. These findings underscore the potential of scSorterDL as an effective and adaptable tool for automated cell type annotation in scRNA-seq research. The code is available on GitHub: https://github.com/kellen8hao/scSorterDL

## Introduction

The advent of single-cell RNA sequencing (scRNA-seq) has greatly advanced research in cellular diagnostics and treatment. Unlike bulk sequencing, which obscures individual cell identities within a mixed population, single-cell sequencing captures and profiles distinct cells within biological samples, especially human tissues, allowing detailed insights into tissue composition and heterogeneity at the gene expression level [1]. By viewing human tissue as an ecosystem composed of diverse cell types, single-cell sequencing uncovers individual cellular characteristics and their dynamic changes, aiding in diagnosing functional disturbances in organs.

Single-cell technology has opened new research opportunities but also introduced the new challenge of “cell type annotation,” a crucial initial step in which cells are labeled according to their gene expression profiles [2]. Accurate annotation is essential for meaningful single-cell analysis, yet it remains challenging, posing a significant analytical hurdle in single-cell genomics. The task of cell type annotation in single-cell RNA sequencing (scRNA-seq) data is inherently complex due to the diverse and variable gene expression patterns across different cell types. Even though single-cell technology is relatively new, various methods have been developed to tackle this challenge. A recent review categorizes these methods into three main approaches: marker gene database-based methods, correlation-based techniques, and supervised classification methods, each offering distinct strengths [3].

As single-cell sequencing technology grows in popularity, the availability of annotated single-cell data has led to the development of numerous cell annotation methods using supervised machine learning. Supervised classification, a well-established approach, utilizes labeled scRNA-seq data for training. Classifiers are trained on reference data with known cell types, then applied to predict cell types in query datasets with similar expression profiles. Many popular machine learning models have been adapted for this purpose: Garnett uses elastic net regression [4], scID employs linear discriminant analysis (LDA) [5], scPred relies on support vector machines (SVMs) [6], and scClassify [7] uses K-Nearest Neighbor (KNN). Ensemble learning combines the classification results of numerous individual models to enhance accuracy and robustness, demonstrating superior performance in cell annotation. For example, SingleCellNet applies random forest classifiers, CaSTLe [8] employs XGBoost, and scAnnotate integrates generative classifiers based on individual gene models [9]. With contemporary single-cell experiments sequencing hundreds of thousands of cells, large reference datasets make deep learning a strong candidate for annotation tools. Examples like ItClust [10], scBERT [11], and scDeepSort [12] show excellent performance on large datasets.

The performance of an annotation tool depends on the machine learning algorithms and other components within its analysis pipeline. To isolate the effect of different machine learning methods, a recent benchmark study compared the most popular algorithms directly, minimizing the impact of other elements in published annotation tools [13]. This study found LDA to be the best overall method. Although LDA does not have the highest accuracy or precision, it is highly competitive and significantly faster than methods with better accuracy metrics. This motivated us to develop a new annotation tool using LDA as the building block. We propose scSorterDL, a novel cell annotation pipeline using LDA as a central building block, with improvements in three key areas. First, we leverage a deep learning framework, known for its effectiveness with large reference datasets. Second, we employ swarm learning, taking advantage of the enhanced performance of ensemble models over individual models. Third, we utilize penalized LDA (pLDA) to mitigate overfitting and collinearity in LDA models without adding notable computational costs.

scSorterDL consists of three modules. The preprocessing module includes standard preprocessing steps for single-cell RNA-seq data, plus a gene screening step to remove irrelevant genes from downstream analysis. The swarm learning module generates a large number of diverse data subsets by randomly removing a large proportion of genes and cells, fitting each subset with pLDA models. Sampling and model fitting are performed on GPUs for high-throughput parallelization. The ensemble module makes preliminary predictions and integrates decisions via weighted voting, implemented as a deep learning architecture with a customized loss function. Both the swarm learning and ensemble modules are built with PyTorch within a unified DNN framework. Using 33 benchmark experiments, we compare scSorterDL against nine popular methods to demonstrate its superior performance.

## Method

We present scSorterDL, a novel method for classifying single-cell RNA-seq data that seamlessly integrates pLDA, swarm learning, and deep learning. Each component brings unique strengths to our approach. Penalized LDA serves as a statistically grounded technique to tackle high-dimensional data challenges through regularization, enhancing the model’s robustness when dealing with many genes relative to the number of samples. Swarm learning boosts the method’s generalizability and resilience by generating multiple diverse data subsets, which are used to train an ensemble of pLDA models. This approach captures different facets of the data, improving predictive performance. Deep learning, employed in the ensemble module, models complex relationships among the outputs of the pLDA models, thus improving classification accuracy by capturing interactions that simpler methods might miss.

**Figure 1:**
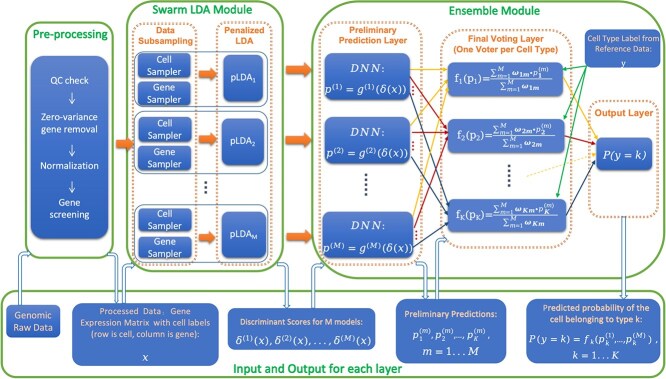
Diagram scSorterDL architecture and workflow. scSorterDL comprises three main components: a data pre-processing module, a Swarm LDA module that generates multiple pLDA models through swarm learning, and an ensemble learning module that combines the outputs of the pLDA models using deep neural networks (DNNs) to generate the final classification decisions.

Figure 1 illustrates the scSorterDL workflow. Below, we provide a detailed explanation of each component in our methodology.

### Pre-processing pipeline

Our data pre-processing pipeline includes quality control (QC) checks, zero-variance gene removal, normalization, and gene screening. First, we filter out low-quality cells and genes with zero variance to ensure the integrity of the data. The normalization step is performed using log-normalization with a scale factor of 10,000 via Seurat [14], which appropriately scales and transforms the gene expression data. Given the large number of genes in single-cell datasets, many are not informative for distinguishing cell types. To address this, we use the Wilcoxon rank-sum test to identify marker genes that are significantly differentially expressed across cell types. For each cell type, the top 400 genes with the lowest p-values are selected as candidate markers. Our empirical analysis indicates that the number of selected genes only affects computational speed, as long as a sufficiently large number of genes is retained.

Additionally, the p-values from the gene screening step are passed to scSorterDL for potential use in the gene sampling component, allowing for more informed sampling strategies if needed.

### Swarm LDA module

The Swarm LDA Module generates multiple random subsets of the original data and fits pLDA models on each subset. The discriminant functions from these pLDA models transform the input genomic data into output scores, which are then used as inputs for the next component, the ensemble layer. This process enables the integration of different models’ insights, enhancing the robustness and accuracy of the final classification.

#### Data subsampling

To introduce meaningful diversity across subsets—which is essential for effective swarm learning—we apply a strategic subsampling approach. Specifically, we sample 80% of the cells and select a number of genes equal to the square root of the total gene count plus seventy. These parameters are configurable and were empirically chosen to balance diversity and biological information retention.

We employ a uniform sampling strategy, ensuring that each gene and cell has an equal chance of being selected. This approach prevents excessive redundancy across subsets and ensures that the pLDA models remain diverse, enhancing the effectiveness of ensembling them. By promoting diversity among the subsets, the penalized models can capture various aspects of the data, enhancing the integration of their results and making the overall analysis more meaningful and robust.

To evaluate diversity introduced by our sampling strategy, we measured overlap ratios across 300 subsampled gene and cell sets using the pancreas dataset (Baron *et al.* [15]). The average gene overlap was only 2.01%, while cell overlap was 66.7%, indicating that the sub-samples exposed pLDA models to highly distinct gene subsets while maintaining biological consistency across cells.

In the supplementary document, we proposed alternative sampling strategies that involve sampling genes and cells with different weights based on their importance. Our empirical experiments show that all sampling strategies have specific strengths, but none consistently outperforms the others.

#### Penalized LDA

LDA has many advantages in cell type classification. It has a simple linear form and closed-form solutions, making it computationally efficient and interpretable. Assuming there are $K$ cell types, LDA projects data from a high-dimensional space (number of genes) into a $(K-1)$-dimensional space, which is suitable for ultra-high-dimensional data with moderate sample sizes. Our empirical analysis shows that vanilla LDA can produce performance non-inferior to many methods specifically designed for cell type annotation using single-cell genomic data.

However, the high dimensionality of gene expression data can lead to singular or ill-conditioned covariance matrices in standard LDA, especially when the number of genes exceeds the number of samples. To address this issue, we use Penalized LDA, an enhanced version that incorporates regularization into the LDA framework. This regularization stabilizes the covariance matrix estimates, enhancing the robustness and applicability of LDA when the number of genes (features) is large relative to the number of samples. Most importantly, Penalized LDA also retains a closed-form solution with linear discriminant functions. Therefore, we choose Penalized LDA as the most essential building block of scSorterDL.

Each random subset generated in the Swarm LDA layer is used to train a Penalized LDA model. Given an outcome vector $y$ (cell type labels) and a gene expression matrix $x$, LDA estimates the class-specific mean vectors $\mu _{k}$ and a shared covariance matrix $\Sigma $, assuming multivariate normality for each class and a common covariance structure across classes. The goal of LDA is to classify new cells based on the discriminant function $\delta _{k}(x)$, which determines the likelihood of the new cell belonging to each cell type $k$.

The standard LDA estimates the parameters $\mu _{k}$ and $\Sigma $ by maximizing the log-likelihood function:


(1) \begin{align*}& \begin{split} log \mathcal{L}(\mu_{1}, \ldots, \mu_{K}, \Sigma) & =-\frac{n}{2} \log |\Sigma| \\ & -\frac{1}{2} \sum_{k=1}^{K} \sum_{i \in C_{k}} (x_{i} - \mu_{k})^{T} \Sigma^{-1} (x_{i} - \mu_{k}), \end{split}\end{align*}

where $n$ is the total number of cells, $C_{k}$ is the set of indices for cells of type $k$, $x_{i}$ is the gene expression vector for cell $i$, $\mu _{k}$ is the mean vector for cell type $k$, and $\Sigma $ is the shared covariance matrix.

Given the high dimensionality of gene expression data, the number of parameters in $\hat{\Sigma }$ can be large, leading to overfitting and numerical instability (e.g. singular covariance matrices). To address these issues, we apply a shrinkage estimator to regularize the covariance matrix, making it closer to a diagonal matrix:


(2) \begin{align*} \hat{\Sigma}(\beta) &= (1-\beta) \hat{\Sigma} + \beta \textrm{Tr}(\hat{\Sigma}) I /p,\end{align*}

where $\hat{\Sigma }$ is the sample covariance matrix and p is the number of genes. The estimator $\hat{\Sigma }(\beta )$ is derived from the penalty $\mathcal{R}(\Sigma ) = \frac{\beta }{2} \left \| \Sigma - \frac{\textrm{Tr}(\Sigma )}{p} I \right \|_{F}^{2}$, which is particularly useful for high-dimensional problems [16].

The penalized LDA’s discriminant function used for classification is given by:


(3) \begin{align*}& \delta_{k}(x) = x^{T} \hat{\Sigma}(\beta)^{-1} \hat{\mu}_{k} - \frac{1}{2} \hat{\mu}_{k}^{T} \hat{\Sigma}(\beta)^{-1} \hat{\mu}_{k} + \log(\pi_{k}).\end{align*}

where $\hat{\mu }_{k}$ is the estimated mean vector for cell type $k$, $\hat{\Sigma }(\beta )$ is the regularized covariance matrix, and $\pi _{k}$ is the proportion of cell type $k$ observed in reference data.

### Ensemble module

The discriminant function (3) processes input genomic information into discriminant scores. With $M$ random subsets generated, the Swarm LDA layer produces $M$ copies of discriminant functions, denoted as $\delta ^{(m)}_{k}(x)$ for $m = 1, \ldots , M$ and $k = 1, \ldots , K$, which provide discriminant scores for each model ($m$) and cell type ($k$).

The ensemble module uses a deep learning approach to aggregate the scores from the swarm of penalized LDA models into final classification decisions. The entire ensemble module can be viewed as a large deep neural network (DNN), composed of multiple components, each of which can itself be a DNN. This architecture allows the model to capture complex relationships among the discriminant scores from different models, improving classification by accounting for interactions that may not be captured by simpler methods. This module consists of two sequential steps that form components of this complex DNN: (1) Preliminary Classification Using Deep Neural Networks: Each individual pLDA model’s discriminant scores are processed by a DNN to produce preliminary predictions (2) Weighted Aggregation Layer: The preliminary predictions from all models are combined using a weighted aggregation layer, which is also implemented as part of the DNN architecture. These steps are described in detail below.

#### Preliminary prediction using a deep neural network (DNN)

The simplest approach to classify a cell from LDA results is to assign it to the cell type $k$ with a probability proportional to the $k$-th discriminant score. In this case, the preliminary predicted probability of the cell belonging to type $k$ is defined as:


\begin{align*} p^{(m)}_{k} \mathop{=}\limits^{\textrm{def}} \Pr(y=k) = \delta^{(m)}_{k}(x)/\sum_{k=1}^{K} \delta^{(m)}_{k}(x). \end{align*}

However, discriminant scores for different cell types may exhibit correlations, either positive or negative, indicating relationships that simple proportionality may not capture effectively. To account for these dependencies, we propose using a complex function $g$ to generate preliminary predictions:


(4) \begin{align*}& \left(p^{(m)}_{1}, \ldots, p^{(m)}_{K} \right) = g^{(m)}\left(\delta_{1}^{(m)}(x), \ldots, \delta_{K}^{(m)}(x)\right),\end{align*}

where a deep neural network (DNN) model is fitted to learn the complex function $g(\cdot )$. The preliminary predictions $p^{(m)}_{k}$ are then used as inputs for the final voting step.

#### Final voting with weighted aggregation

In the final voting step, we combine the preliminary predictions $p^{(m)}_{k}$ from each pLDA model using a weighted aggregation approach. The goal is to assign a cell to a cell type based on a weighted average of the predicted probabilities from all models, where the weights are specific to each cell type and model combination.

Let $w_{mk}$ denote the weight for the $m$-th pLDA model’s prediction for cell type $k$. The final predicted probability of the cell belonging to type $k$ is given by:


(5) \begin{align*}& P(y = k) = f_{k} \left( p^{(1)}_{k}, \ldots, p^{(M)}_{k} \right) = \sum_{m=1}^{M} w_{mk} \cdot p^{(m)}_{k}/\sum_{m=1}^{M} w_{mk}.\end{align*}

To learn the weights $w_{mk}$, we use a softmax function to ensure that the weights are positive and sum to one for each cell type. The softmax approach allows the model to automatically learn the optimal contribution of each pLDA model to the final prediction for each cell type, adapting to the strengths and weaknesses of individual models. This weighted voting scheme improves the robustness of the classification by giving more importance to models that are better suited for predicting specific cell types.

#### Parameter estimation and training

We consider the $M$ DNNs, $g^{(m)}$, as components of a larger network, with the softmax voting function $F$ serving as the final layer of this network. The model parameters are learned jointly using a combined loss function for the entire ensemble layer:


(6) \begin{align*}& \begin{split} \mathcal{L}(x, y) = \alpha \mathcal{C}\left(F\left(g^{(1)}\left(\delta(x)\right), \ldots, g^{(M)}\left(\delta(x)\right)\right), y\right) \\ + (1-\alpha) \sum_{m=1}^{M} \mathcal{C}\left(g^{(m)}\left(\delta(x)\right), y\right), \end{split}\end{align*}

where $F$ represents the output of the final voting layer, $g^{(m)}$ is the preliminary prediction from each pLDA model, and $\mathcal{C}$ denotes the cross-entropy loss function.

Note (6) is a customized loss function, which aligns the DNN outputs of preliminary predictions with the final decision. This mapping guarantees consistent cell type alignment across all models. In contrast, the standard loss function for DNN only focuses on the output of the final layer, which corresponds to set $\alpha =1$ in our loss. Our customized loss function introduces an additional component, effectively creating a skip connection from the intermediate outputs of the DNN to the final decision. This approach is similar to traditional skip connections in residual networks. To optimize the network, we experimented with several optimization algorithms, including Stochastic Gradient Descent (SGD) [17, 18], NAdam [19], and AdamW [20]. We found that SGD performed the best on our reference datasets.

### Software implementation

When implementing our algorithm, we aimed to maximize the benefits of GPU computing to enhance the performance of our code. While it is common practice to utilize GPUs for training deep neural networks in the ensemble layer, we extended GPU utilization to the Swarm LDA layer as well. The Swarm LDA layer involves generating a large number of random subsets and fitting penalized LDA models on each subset using closed-form solutions. Although each individual pLDA model is computationally simple to fit, the sheer number of models required makes this step computationally intensive.

GPUs are particularly well-suited for this type of workload due to their ability to parallelize thousands of computation tasks simultaneously, far exceeding the parallelization capabilities of standard CPUs. By leveraging the parallel processing power of GPUs, we significantly accelerated the computation of the Swarm LDA layer, allowing for efficient training even with a large number of pLDA models. We implemented the Swarm LDA computations using GPU-compatible operations (nn.module) in PyTorch with a closed-form solution, ensuring that both the data sampling and the pLDA model fitting steps benefit from GPU acceleration. This allows us to treat each LDA as an NN layer that is easily composed with other NN layers and integrated into other NN architectures. This holistic use of GPU resources across both the Swarm LDA and ensemble layers contributed to the overall efficiency and scalability of scSorterDL.

Following the architecture discussed above, our implementation consists of several key components: Samplers component (implemented in samplers.py), Discriminant Analysis component( da.py which implements both LDA and QDA), the ensemble component (scSorterDL.py), and an integrator component (train.py) that brings all these elements together. All components leverage PyTorch [21] to perform their functionalities. Specifically, PyTorch tensors and functions are used extensively across all scripts to support both CPU and GPU implementations. To maximize efficiency, we have designed minimal data transfer between the CPU and GPU. The samplers can run directly on the GPU if the user chooses to.

Although scSorterDL can run on CPUs, we recommend using GPUs for computational efficiency, especially when dealing with large datasets. In our experiments, we utilized the Alliance clusters (https://docs.alliancecan.ca/), each equipped with a minimum of 32 cores, 128,GB of RAM, and four Nvidia GPUs with at least 16,GB of memory. We provided shell scripts and Slurm job submission scripts to automate the process, which can be adjusted by users to match their computational environment.

By highlighting our extensive use of GPUs not only in the ensemble layer but also in the Swarm LDA layer, we emphasize the computational efficiency and scalability of scSorterDL. Leveraging GPUs for both layers allows us to handle the computational demands of generating and fitting a large number of pLDA models, fully utilizing the parallel processing capabilities of modern hardware.

## Performance evaluation

### Experimental design, datasets, and comparative methods

We evaluated the performance of scSorterDL against 9 other popular methods using real scRNA-seq datasets encompassing a wide variety of tissue compositions, sequencing protocols, and species. Our evaluation mimics two of the most common real-world scenarios encountered when annotating cell types in scRNA-seq data.

In the first scenario, we assume that a published dataset with annotated cell types is available, closely matching all characteristics of the data to be annotated. This situation represents the ideal case where the reference data perfectly aligns with the target data in terms of species, tissue composition, and sequencing protocol. To mimic this scenario, we conducted experiments on 13 real datasets. We perform cross-validation within each dataset where the species, tissue composition, and sequencing protocol are consistent. This approach tests how well scSorterDL performs when both reference and query data come from the same source. Detailed information about 13 datasets are provided in Table 1.

**Table 1:** Datasets used for cross-validation experiments

Datset No.	Study	Organism and Tissue	Sequence Platform	No. of cells
1	Baron *et al.* [15]	Human pancreas	inDrop	8562
2	Muraro *et al.* [22]	Human pancreas	CEL-seq2	2285
3	Segerstolpe *et al.* [23]	Human pancreas	SMART-Seq2	2394
4	Xin *et al.* [24]	Human pancreas	SMARTer	1492
5	Tasic *et al.* [25]	Mouse primary visual cortex (PVC)	SMARTer	1727
6	Campbell *et al.* [26]	Mouse HArc-ME	Drop-seq	20921
7	Ding *et al.* [27]	Human PBMC	10x Chromium (v2)	3362
8	Schaum *et al.* [28]	Whole Mus musculus	SMART-Seq2	24622
9	Zheng *et al.* [29]	FACS-sorted PBMC	10X CHROMIUM	91649
10	Zheng *et al.* [29]	Human PBMC	10X CHROMIUM	2467
11	Tasic *et al.* [30]	Mouse neocortex	SMART-Seq	3500
12	Tian *et al.* [31]	Mixture of five human cancer cell lines	CEL-seq2	909
13	Tian *et al.* [31]	Mixture of five human cancer cell lines	10X CHROMIUM	3918

In the second scenario, we explore a more complex situation in which we have access to annotated data that only partially aligns with our scRNA-seq data. This includes datasets from the same species and tissue types but collected using different sequencing protocols. To simulate this scenario, we set up 20 experiments aimed at evaluating external validation with paired datasets. Each experiment consists of independent reference and query datasets that share the same species and tissue types while differing in their sequencing protocols. This configuration mirrors real-world instances where only partially corresponding annotated datasets are accessible. We examined both UMI-based protocols, such as 10X Chromium, inDrop, and Drop-seq, alongside full-length transcript-based protocols like SMART-Seq2. This comprehensive approach enables us to thoroughly evaluate the generalizability of each method under varying technical conditions. For detailed information about the dataset pairs used in these 20 experiments, please refer to Table 2.

**Table 2:** Dataset pairs used for evaluation of cross-platform annotation

Reference Data				Query Data			
Study	Organism and Tissue	Sequence Platform	No. of cells	Study	Organism and Tissue	Sequence Platform	No. of cells
Baron *et al.* [15]	Human pancreas	inDrop	8562	Xin *et al.* [24]	Human pancreas	SMARTer	1492
Muraro *et al.* [22]	Human pancreas	CEL-seq2	2285	Xin *et al.* [24]	Human pancreas	SMARTer	1492
Campbell *et al.* [26]	Mouse HArc-ME	Drop-seq	20921	Tasic *et al.* [25]	Mouse primary visual cortex	SMARTer	2285
Ding *et al.* [27]	Human PBMC	10x(v2)	3362	Ding *et al.* [27]	Human PBMC	10x(v3)	3222
						Drop-seq	6584
						inDrops	6584
Ding *et al.* [27]	Human PBMC	10x(v3)	3222	Ding *et al.* [27]	Human PBMC	CEL-Seq2	526
						Smart-seq2	526
Ding *et al.* [27]	Human PBMC	Drop-seq	6584	Ding *et al.* [27]	Human PBMC	10x (v2)	3362
						Seq-Well	3727
Ding *et al.* [27]	Human PBMC	inDrops	6584	Ding *et al.* [27]	Human PBMC	10x (v2)	3362
						10x(v3)	3222
						Drop-seq	6584
						CEL-Seq2	526
						Smart-seq2	526
Ding *et al.* [27]	Human PBMC	CEL-Seq2	526	Ding *et al.* [27]	Human PBMC	Smart-seq2	526
Ding *et al.* [27]	Human PBMC	Smart-seq2	526	Ding *et al.* [27]	Human PBMC	CEL-Seq2	526
Schaum *et al.* [28]	Whole Mus musculus	SMART-Seq2	24622	Schaum *et al.* [28]	Whole Mus musculus	10x	20000
Schaum *et al.* [28]	Mus Lung	SMART-Seq2	1563	Schaum *et al.* [28]	Mus Lung	10x	1303
Zheng *et al.* [29]	PBMC	10X	91649	Zheng *et al.* [29]	PBMC	10x	2467

In total, we conducted 33 experiments across these two scenarios. The datasets used in these experiments were obtained from 13 published studies, with some studies providing multiple datasets. The studies we source our datasets are as follows. For mouse brain datasets, we used the Primary Visual Cortex (PVC) and Neocortex (VISp and ALM) datasets by Tasic *et al.* [25, 30], as well as the HArc-ME dataset by Campbell *et al.* [26]. We included the mouse pancreas dataset from Baron *et al.* [15]. For human pancreas datasets, we utilized datasets from Baron *et al.* [15], Muraro *et al.* [22], Xin *et al.* [24], Wang *et al.* [32], and Segerstolpe *et al.* [23]. The human PBMC datasets were obtained from Ding *et al.* [27] and Zheng *et al.* [29]. We also included the Tabula Muris dataset from The Tabula Muris Consortium (Schaum *et al.*) [28]. Additionally, we used the 10X and CEL-Seq2 datasets from Tian *et al.* [31], based on five human lung cancer cell lines, referred to as the CellBench datasets. These datasets provide a comprehensive collection that includes various species (mouse and human), tissue types (brain, pancreas, PBMCs, lung cancer cell lines), and sequencing protocols (e.g. 10X Genomics, CEL-Seq2), allowing us to demonstrate the robustness and applicability of scSorterDL in diverse scenarios. To contextualize the evaluation, we also summarized cell counts, cell type distributions, and gene sparsity across all datasets(Table S1). These statistics reveal substantial variation in size and complexity, including imbalanced cell type distributions and subpopulations with fewer than 10 cells. Detailed information, including accession numbers, sequencing platforms, number of genes, and number of cell types, can be found in Supplementary Table S6(on GitHub).

In each experiment, we compared our scSorterDL model against nine publicly available cell-type annotation methods: Seurat [14], scmap [33], SingleR [34], CHETAH [35], SingleCellNet [36], scID [5], scClassify [7], CaSTLe [8], and SCINA [37]. These popular tools are developed specifically for automated single-cell scRNA-seq annotation and are frequently referenced in the literature as benchmarks for evaluating new cell annotation methods. When applying other methods in our comparisons, we follow the parameter settings used in the benchmark experiment of a recently published study [38].

The most commonly used criterion for evaluating cell annotation methods is overall accuracy, defined as the proportion of cells correctly assigned to their respective cell types. We report overall accuracy as the primary focus in our evaluation, while also including the F1 score (Figure S1) in the supplementary materials can provide additional insight by accounting for both precision and recall in classification.

### Comparison results

#### Cross validation results

**Figure 2:**
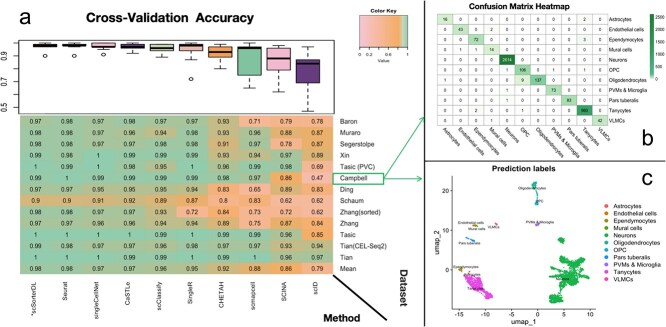
Evaluation of performance in the cross-validation scenario: Panel (a) displays the performance of scSorterDL compared with nine other cell-type annotation methods under cross-validation. In the heatmap, rows correspond to 13 experiments (same order as in Table 1), with the bottom row showing each method’s average accuracy across all experiments. Columns represent the 10 methods, arranged from left to right by their average overall accuracy. A boxplot above the heatmap summarizes each method’s accuracy across the 13 experiments. Panel (b) shows a heatmap of the confusion matrix for scSorterDL on the Campbell *et al.* dataset, with rows and columns representing reference and predicted cell labels, respectively. Each cell in the matrix displays the counts of a specific query cell type assigned to a particular reference cell label. Panel (c) visualizes cells from the Campbell *et al.* dataset in two UMAP dimensions, with color indicating the scSorterDL-predicted cell type labels.

Figure 2a shows the overall accuracy of all the methods, ranging from $0.98$ to $0.79$. The heatmap displays the accuracy of each method (columns) on each dataset (rows), with the last row indicating the average accuracy of each method across all 13 experiments. Each box in the boxplots summarizes a method’s accuracy across the 13 experiments, with methods ordered from left to right by their average performance. Seurat and scSorterDL emerge as the top performers, both achieving the highest average overall accuracy of $0.98$, with the lowest variance in performance, highlighting their robustness.

While Fig. 2a provides an overall summary of annotation performance across all cell types, Fig. 2b and c present a more detailed evaluation of specific cell types within a single experiment. Figure 2b displays the confusion matrix of the classification results for each cell type, revealing that scSorterDL achieves near-perfect annotation accuracy, even for rare cell types with limited cell numbers. Figure 2c visualizes the scRNA-seq data from the mouse hypothalamic arcuate-median eminence complex (HArc-ME) dataset [26] in two dimensions using UMAP dimension reduction.

#### Cross-platform results

**Figure 3:**
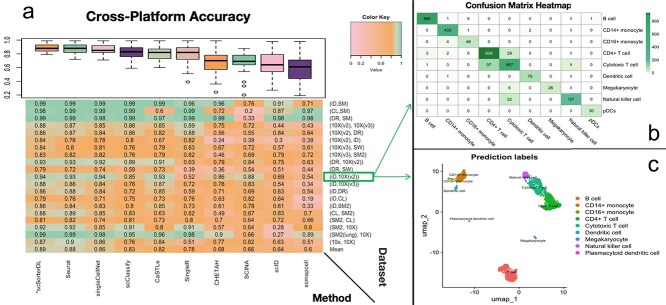
Performance Evaluation of Cross-Platform Annotation: Reference and query datasets are well matched, differing only in the protocols used for their generation (rows displayed in the same order as in Table 2. Results are summarized in the same format as Fig. 2, but row names show the pair of two protocols in reference and query sets. The protocol names are abbreviated as iD = inDrops; CL = CEL-Seq2; SM = SMARTer; SM2 = Smart-seq2; DR = Drop-seq; 10X(v2) = 10x Chromium (v2); 10X(v3) = 10x Chromium (v3); SW = Seq-Well. Panels (b) and (c) showing scSorterDL’s detailed annotation performance on human PBMC datasets, where the reference dataset was profiled by inDrops and the query dataset by 10X(v2).

Figure 3 summarizes the cross-platform annotation performance of methods in a consistent way as what’s given in Fig. 2. Overall, we observed a similar pattern in the performance rankings of these approaches. The scSorterDL shows excellent performance and robustness by outperforming other methods with the highest average overall accuracy (0.89) and smallest performance variance across 20 experiments. Figure 3c illustrate that cells of different types can be in close proximity within the PBMC data (inDrop as the reference and 10X Genomics as the query). Despite the high similarity in gene expression profiles among certain cell types, Fig. 3b shows only a few misclassifications were observed, even for cell types located very close to each other.

In additional mixed-platform experiments covering combinations of major scRNA-seq technologies such as inDrop, CEL-seq2, SMART-seq2, SMARTer, 10X Chromium (v2/v3), and Drop-seq (as described in the Supplementary Information), scSorterDL achieved a mean accuracy of 0.98 in cross-validation and 0.91 in cross-platform settings, consistently ranking first among all evaluated methods (Table S2, S3).

In parallel with the accuracy comparison, scSorterDL also outshines other methods in terms of F1 scores (Supplementary Figure S1). Although Seurat also performs well, scSorterDL demonstrates a statistically significant improvement (p = 0.0347) based on a paired t-test comparing two vectors of F1 scores, each generated by Seurat and scSorterDL across 20 individual datasets. Unlike Seurat, which relies on unsupervised clustering followed by anchor-based labeling, scSorterDL is better equipped to handle large-scale datasets due to its GPU-accelerated processing. Seurat struggles in cases where anchor pairs are sparse, particularly for rare cell types, leading to misclassifications. This limitation is evident in challenging datasets such as those in cancer studies, where rare cell populations lack sufficient neighborhood information for accurate annotation. In contrast, scSorterDL employs a balanced cell sampling strategy, ensuring rare cell types are well represented and leading to more reliable predictions in these scenarios.

When reference and query sets are generated using different protocols, most experiments show an expected accuracy drop compared to cross-validation experiments. However, no accuracy drop is observed in two pairs of pancreas-related datasets (first two rows of the heatmap in Fig. 3a), suggesting gene expression profiles of human pancreas cell types may be sufficiently distinct to support accurate annotation across platforms.

The Schaum *et al.* dataset poses a particular challenge in both scenarios due to its numerous cell types, some with very few cells, and the high similarity of gene expression profiles across multiple cell types. Cross-validation experiments using this dataset yielded relatively low accuracy for many methods. Similarly, cross-platform experiments, where 37 cell types profiled in full-length Smart-seq2 as the reference and 32 cell types profiled with 10X Genomics as the query, led to poor results for several methods. Despite these challenges, scSorterDL consistently performs well, outperforming other methods on this dataset, and shows no accuracy drop even with cross-platform noise. This result highlights the strength of our approach in handling complex scenarios with highly similar cell populations.

## Discussion and conclusion

**Broader Applicability of the scSorterDL Framework:** Although scSorterDL is specifically designed for cell type annotation using scRNA-seq data, its underlying framework can be adapted to various machine learning and deep learning applications. This robust approach manages high-dimensional data (e.g. gene expression) and large datasets (e.g. single cells) by combining swarm learning and deep learning architectures, with random sampling of features and samples on GPUs. This dual strategy leverages the strengths of both components: swarm learning integrates a diverse ensemble of parsimonious models, ensuring classification robustness, while the deep learning component flexibly combines LDA results for an optimized outcome. In our evaluation, we included scmap as a benchmark due to its well-established robustness in low-cell-count scenarios. This makes it a particularly meaningful comparator when assessing performance on sparse or imbalanced datasets.

**Variations of scSorterDL’s Components:** The scSorterDL network comprises multiple components, each with potential variations. We explored five alternatives. (1) The supplementary documents describe four different samplers in the swarm LDA layer, sampling genes and cells with varying weights based on importance. Our preliminary experiments show that each strategy has strengths in specific datasets, with no clear overall winner. (2) We evaluated an alternative regularized estimator using Tikhonov regularization, $\mathcal{R}(\Sigma ) = \frac{\beta }{2} \|\Sigma - I\|_{F}^{2}$ [39], yielding a penalized covariance estimator, $\hat{\Sigma }(\beta ) = \hat{\Sigma } + \beta I$. This did not outperform our default choice (2), which also provides a closed-form solution, making it our recommended default. (3) We tried replacing softmax-weighted voting functions $f$ with a more complex DNN. Although this can provide a meaningful improvement with huge training datasets, it significantly increases training time and memory requirements. For instance, in a PBMC experiment (10x Genomics as reference), the DNN voter improved accuracy from 0.87 to 0.89 with over 90,000 cells in the reference set. Our software allows users to use DNN voters but recommends them only for huge reference datasets. (4) In the final voting layer, scSorterDL uses cell-type-specific voters ($f_{1}, \ldots , f_{K}$). We tested a common voter ($f$) across all cell types (i.e. setting $f_{1} = \dots = f_{K} = f$), which resulted in poorer performance in our preliminary results. Supplementary Figure S2. shows heatmaps of scSorterDL’s weights estimated for different cell types, which appear distinct, confirming the necessity of cell-type-specific voters. (5) We have compared scSorterDL’s customized loss function (6) in the Ensemble module with the standard loss, which only focuses on the output of the final layer (equivalent to the setting $\alpha =1$ in our customized loss). For instance, the standard loss function reduces the accuracy of scSorterDL from 0.87 to 0.847 in the PBMC experiment mentioned above.

**Limitations and Future Work:** (1) scRNA-seq data initially contains tens of thousands of genes, reduced here to a few thousand by screening the top 400 genes for each cell type. Performance could improve with alternative dimension reduction methods. We plan to replace the current screening with an autoencoder with a customized loss function and integrate it with scSorterDL’s deep learning network into an end-to-end DNN architecture, minimizing preprocessing reliance. (2) We evaluated scSorterDL on six challenging cancer datasets and found that it maintained strong performance despite tumor heterogeneity, achieving a mean accuracy of 0.88 and ranking 2nd among all methods (Table S4, S5). Several baseline methods failed to complete these tasks due to memory or marker limitations, which we report transparently. (3) While scSorterDL performs well on large datasets, it requires substantial training time and memory and may yield less reliable results with smaller datasets. Future work will explore meta- and transfer-learning frameworks: meta-learning across diverse and extensive scRNA-seq datasets (e.g. cell atlases) will develop a robust pre-trained model, while transfer-learning will allow fine-tuning the final layers of network in user-specific datasets, reducing sample requirements and computational demands for specialized datasets. (4) Expanding performance evaluation to include cross-species annotation (e.g. reference on mouse samples and query on human samples) is planned, as this task presents unique challenges. (5) Real scRNA-seq data may contain cell types absent from the reference set. We aim to address this by extending scSorterDL with a semi-supervised learning flavour. (6) Due to high computational demands, scSorterDL’s hyperparameter tuning was performed through empirical cross-validation in a subset of datasets. We explored candidate values of the penalty parameter ($\beta =0.1, 0.01, 0.000001$) for pLDA and settled on $0.000001$. We explored the number of swarm LDAs ($5, 10, 50, 100, 300, 500, 600, 1000, 1200, 1600, 2000$) and settled on $300$. As scSorterDL gains adoption, we will optimize its hyperparameters using a more comprehensive approach to improve its performance further. Moreover, to ensure reliable gene selection and robust classification performance in scSorterDL, we recommend using at least 20–30 cells per cell type. This guideline is consistent with prior studies [40, 41] that highlight the importance of statistical stability in differential expression analysis and cell type annotation, particularly in the context of sparse and high-dimensional single-cell RNA-seq data.

**Conclusion:** In this study, we introduced scSorterDL, a novel framework that integrates pLDA, swarm learning, and deep neural networks for cell type annotation in single-cell RNA sequencing data. By generating diverse data subsets and fitting multiple pLDA models, scSorterDL captures various aspects of the high-dimensional and sparse scRNA-seq data. The ensemble module uses deep learning to model complex relationships among the outputs of the pLDA models, enhancing classification accuracy by accounting for interactions that may be overlooked by traditional methods. The swarm and ensemble modules are integrated into a DNN architecture, fully utilizing high-throughput GPU parallelization. Our extensive evaluations across 33 experiments, including cross-validation within datasets and cross-platform validations using independent datasets, demonstrate that scSorterDL outperforms nine popular cell annotation tools in both overall average accuracy and robustness. Even in challenging scenarios involving a large number of cell types with highly similar gene expression profiles, scSorterDL shows exceptional performance.

**Key Points**

- scSorterDL integrates penalized Linear Discriminant Analysis (pLDA), swarm learning, and deep neural networks (DNNs) to achieve superior classification performance by capturing both linear and nonlinear relationships within high-dimensional scRNA-seq data.
- By generating diverse random subsets of the data and fitting pLDA models to each subset, scSorterDL mitigates dataset-specific biases and enhances the generalizability of cell type classification across varying experimental conditions.
- The ensemble module leverages a deep neural network to capture complex dependencies in pLDA scores, revealing biological patterns missed by traditional models.
- Penalized LDA mitigates overfitting and collinearity, enhancing model stability and interpretability in high-dimensional scRNA-seq datasets.
- GPU-accelerated swarm learning and deep learning enable efficient processing of large-scale single-cell datasets for high-throughput applications.

## Supplementary Material

BIB__scSorterDL_supp_bbaf446
