# Supplementary material for: scSorterDL: a deep neural network-enhanced ensemble LDAs for single cell classifications
Source: Brief Bioinform. 2025 Sep 1;26(5):bbaf446. doi: 10.1093/bib/bbaf446 (PMC12400813; doi:10.1093/bib/bbaf446)
Supplement: BIB__scSorterDL_supp_bbaf446 [file bib__scsorterdl_supp_bbaf446.pdf]

Supplemetary Document - scSorterDL: A Deep Neural Network-Enhanced Ensemble LDAs for Single Cell Classifications

Kailun Bai[1], Belaid Moa[2], Xiaojian Shao[3,4], and Xuekui Zhang[1]*

[1] Department of Mathematics and Statistics, University of Victoria, Victoria BC, Canada
[2] Digital Research Alliance of Canada, Victoria BC, Canada
[3] National Research Council Canada, Ottawa ON, Canada
[4] Ottawa Institute of Systems Biology, Department of Biochemistry, Microbiology and Immunology, University of Ottawa, Ottawa ON, Canada

S1 F1 Score Comparison results for Cross Platform Experiments

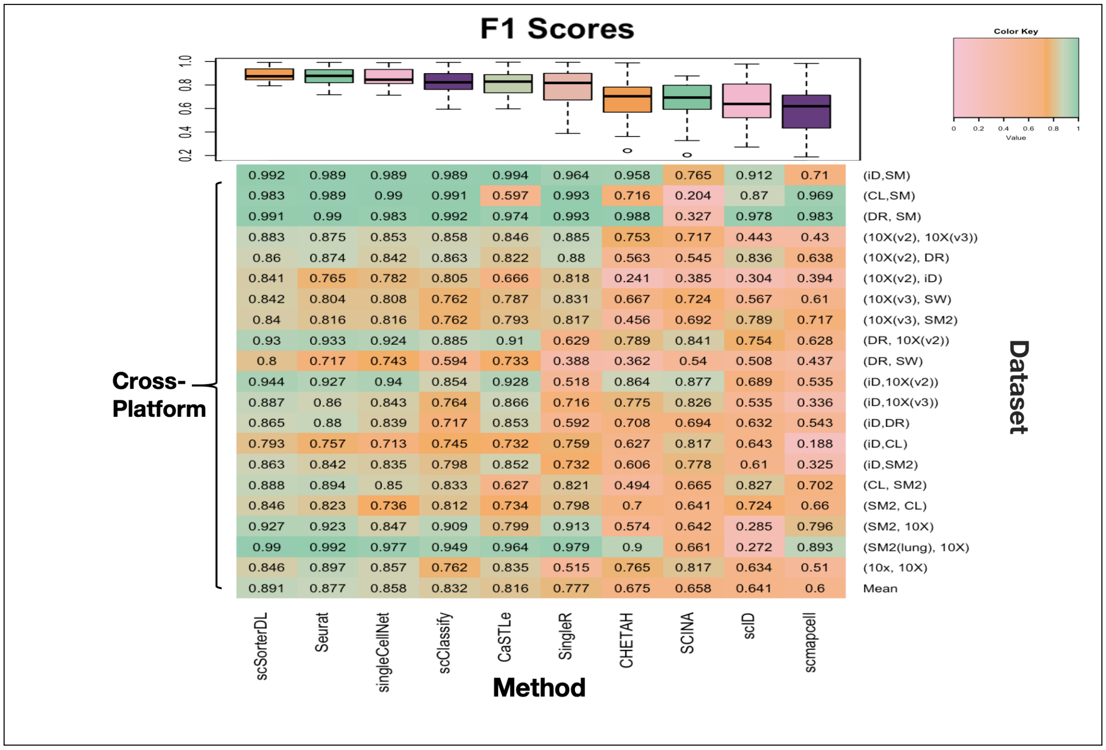

Fig. S1: Cross Platform F1 Scores.

FigureS1 shows the Micro F1 Score of scSocrterDL and 9 competing methods in the cross-platform scenario. The methods were sorted by their average performance across the datasets, ranging from an average F1 Score of 0.891 to 0.600.

* Corresponding authors: xuekui@uvic.ca

S2 Alternative Sampling strategies for Swarm LDA layer

Significance-Based Sampling. This method leverages gene significance scores obtained from a screening step to guide the sampling process. The significance of each gene, assessed using $-(log_{10}(p-value))$ for distinguishing cell types, is used to construct a sampling distribution that prioritizes more important genes. This approach increases the likelihood of selecting biologically relevant genes, while still incorporating some randomness. The significance scores are based on the probabilities derived from the gene significance information, and the sampling probabilities are adjusted using a multinomial sampling method, which determines the degree of bias towards significant genes.

Sparse Gene Sampling. Sparse gene sampling employs Dirichlet distributions to generate sparse sampling distributions, where some genes have a much higher probability of being selected than others. Different concentration parameters of the Dirichlet distribution can be used to control the level of sparsity, allowing us to sample a subset where only a few genes dominate. This approach is beneficial in cases where a small number of genes are believed to carry most of the relevant information, but it is sensitive to the choice of hyperparameters.

Balanced Cell Sampling. To address the issue of class imbalance, which is common in single-cell datasets, balanced cell sampling increases the probability of selecting cells from rare cell types. The sampling weights are inversely proportional to the abundance of each cell type in the original data, enhancing the representation of underrepresented classes in each subset. The reweighting factor is set by drawing samples based on a multinomial distribution, using the defined probabilities, and selecting the specified number of samples without replacement. This allows the method to adjust the emphasis placed on rare cell types and ensure a more balanced sample distribution.

Stratified Cell Sampling. Stratified sampling ensures that the sampled cells follow the same distribution as the original dataset, maintaining the proportions of each cell type. This method aims to preserve the dataset's structure in the sampled subsets, avoiding any distortion of the natural class distribution. The number of cells sampled from each cell type is determined based on the proportion of each cell type in the original dataset, which matches the original class ratios in the dataset.

User-Defined Sampling. The tool also supports custom sampling distributions for both genes and cells, allowing users to specify their own sampling strategies based on the characteristics of their datasets or specific analytical needs.

Our investigation showed that some strategies performed better than others in specific scenarios. For example, stratified sampling did not yield the improvements we initially expected, while balanced cell sampling was effective for improving the representation of rare cell types. Sparse gene sampling was highly sensitive to the Dirichlet hyperparameters, impacting its robustness. Although significance-based sampling showed promise, we plan to explore it further in future work. To demonstrate the tool's functionality, we chose to use uniform sampling for both genes and cells.

S3 Fine-grain scSorterDL shallow weights for 1000 LDAs for each cell type in the case of PBMC dataset.

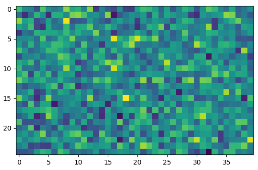

(a) cell type 1.

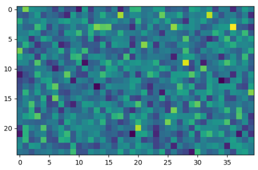

(b) Cell type 2.

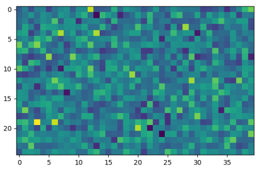

(c) Cell type 3.

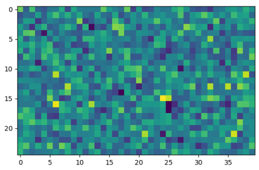

(d) Cell type 4.

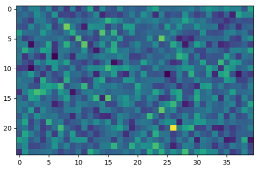

(e) Cell type 5.

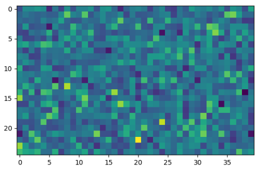

(f) Cell type 6.

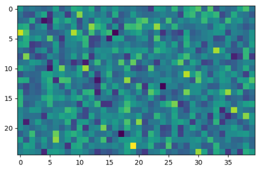

(g) Cell type 7.

Fig. S2: Weights of scSorterDL's final voting layer 1000 LDAs for each cell type in the case of PBMC dataset. Heatmaps of different cell types look very different, supporting to use cell type-specific voters in the voting layer.

Figure S2 shows weights of scSorterDL's final voting layer 1000 LDAs for each cell type in the case of PBMC dataset. It illustrates how weights differ for each cell type. Each square represents an LDA, with color indicating its relative importance in the final output. The substantial variation in LDA performance across cell types highlights the need for tailored weighting strategies to enhance classification accuracy and precision. This emphasizes the importance of cell type-specific weighting and the necessity for better architectures.

Table S1: Summary of datasets used in this study, including cell count, number of annotated cell types, and mean zero gene rate

Dataset	No. of Cells	No. of Cell Types	Mean Zero Gene Rate
Baron	8,569	14	0.906
Muraro	2,285	13	0.846
Segerstolpe	2,394	13	0.819
Xin	1,492	4	0.858
Tasic	1,727	6	0.689
Campbell	20,921	11	0.928
Ding (10x v2)	3,362	9	0.966
Schaum (SMART-Seq2)	24,622	37	0.866
Zheng (FACS-sorted)	91,649	7	0.968
Zheng (10x)	2,467	6	0.940
Tasic (2018)	3,500	9	0.835
Tian (CEL-Seq2)	909	5	0.577
Tian (10x)	3,918	5	0.630
Ding (10x v3)	3,222	8	0.955
Ding (CEL-Seq2)	6,584	9	0.975
Ding (Drop-seq)	6,584	9	0.985
Ding (inDrops)	3,727	7	0.984
Ding (Seq-Well)	526	7	0.924
Ding (SMART-seq2)	526	7	0.922
Schaum (10x)	20,000	32	0.904
Schaum (Lung SMART-Seq2)	1,563	10	0.853
Schaum (Lung 10x)	1,303	8	0.883

S4 Dataset Summary

To ensure robust benchmarking across diverse biological and technical conditions, we compiled 22 publicly available single-cell RNA-seq datasets. These datasets vary in cell count, tissue origin, number of annotated cell types, and sequencing platform. In addition to biological diversity, we also examined technical variation, particularly sparsity, by calculating the mean proportion of genes with zero expression per cell (Mean Zero Gene Rate). This metric reflects dropout levels and overall data quality. Table S1 summarizes key statistics for all datasets used in our benchmarking pipeline.

Table S2: Accuracy from cross-validation on mixed-platform single-cell RNA-seq datasets (validation setting)

Dataset(mixed-platform)	`scSorterDL`	singleCellNet	SingleR	scClassify	CHETAH	scmap-cell	scID	SCINA	CaSTLe	Seurat
inDrop + CEL-seq2	0.97	0.97	0.97	0.97	0.93	0.88	0.40	0.66	0.11	NA
inDrop + SMART-seq2	0.98	0.97	0.97	0.97	0.94	0.91	0.40	0.71	0.10	NA
CEL-seq2 + SMART-seq2	0.98	0.97	0.98	0.96	0.96	0.96	0.70	0.50	0.10	NA
CEL-seq2 + SMARTer	0.98	0.98	0.97	0.96	0.95	0.93	0.85	0.43	0.07	NA
SMART-seq2 + SMARTer	0.98	0.98	0.98	0.97	0.94	0.95	0.55	0.63	0.05	NA
10x(v2) + 10x	0.96	0.96	0.94	0.94	0.87	0.56	0.39	0.31	0.21	NA
Mean	0.98	0.97	0.97	0.96	0.93	0.86	0.55	0.54	0.11	NA

Table S3: Accuracy from cross-platform classification on mixed-platform single-cell RNA-seq datasets

Dataset(mixed-platform)	`scSorterDL`	singleCellNet	SingleR	scClassify	CHETAH	scmap-cell	Seurat	SCINA	scID	CaSTLe
inDrop + CEL-seq2	0.96	0.99	0.99	0.82	0.99	0.89	0.32	0.10	0.07	NA
inDrop + CEL-seq	0.97	0.96	0.98	0.97	0.95	0.84	0.71	0.75	0.44	0.01
SMART-seq2 + inDrop	0.98	0.99	0.99	0.33	0.99	0.92	0.26	0.07	0.23	NA
SMART-seq2 + inDrop	0.97	0.94	0.97	0.97	0.93	0.88	0.13	0.77	0.28	0.12
SMART-seq2 + CEL-seq2	0.99	0.99	0.99	1.00	0.96	0.97	0.54	0.05	0.12	NA
SMART-seq2 + CEL-seq2	0.96	0.87	0.96	0.92	0.55	0.87	0.64	0.25	0.70	0.11
10X(v2) + Drop-seq	0.83	0.79	0.55	0.80	0.53	0.19	0.21	0.21	0.10	0.16
10X(v2) + inDrop	0.86	0.84	0.79	0.84	0.82	0.02	0.39	0.21	0.17	0.11
SMART-seq2 + 10X(v3)	0.84	0.80	0.82	0.76	0.78	0.55	0.48	0.52	0.24	0.15
10X(v2) + inDrop	0.80	0.83	0.76	0.82	0.68	0.06	0.26	0.04	0.19	0.21
Mean	0.91	0.90	0.88	0.82	0.82	0.62	0.39	0.30	0.25	0.13

S5 Benchmarking on Mixed-Platform Single-cell RNA-seq Datasets

To address platform heterogeneity in real-world applications, we performed extensive mixed-platform evaluations using the Pancreas and PBMC datasets included in the main paper. In total, we conducted 16 experiments—6 mixed-platform validation tasks and 10 cross-platform classification tasks—spanning combinations of major scRNA-seq technologies, including inDrop, CEL-seq2, SMART-seq2, SMARTer, 10X Chromium (v2/v3), and Drop-seq. These experiments were designed to assess the cross-platform robustness of `scSorterDL` in comparison to nine competing annotation tools.

Table S2 summarizes the accuracy results from the six within-dataset mixed-platform validation tasks. Table S3 shows the accuracy from ten cross-platform classification tasks, in which training and testing were performed across datasets with non-overlapping sequencing protocols. Several baseline methods, including Seurat and CaSTLe, failed on multiple tasks due to expression scale mismatches or internal prediction errors, and are marked as “NA”.

Table S4: Benchmarking accuracy across methods on Cancer Datasets

Dataset	CaSTLe	Seurat	scSorterDL	singleCellNet	scClassify	SingleR	scmap-cell	SCINA	CHETAH	scID
Kim	NA	0.87	0.85	NA	NA	0.86	0.77	0.69	NA	NA
Zilionis	0.80	0.78	0.79	0.79	0.74	0.63	0.71	0.43	0.53	0.45
Wu	0.98	0.99	0.97	0.97	0.98	0.94	0.96	0.68	0.95	0.69
Liu	0.94	0.95	0.91	0.94	NA	0.88	0.74	0.74	0.75	0.52
PanglaoDB	0.91	0.93	0.87	0.91	0.83	0.82	0.71	NA	0.35	NA
PanglaoDB(2)	0.86	0.86	0.88	0.80	0.70	0.66	0.47	NA	0.34	NA
Mean	0.90	0.90	0.88	0.88	0.81	0.80	0.73	0.64	0.58	0.55

Table S5: Details of cancer datasets used for benchmarking

Study	Organism and Tissue	No. of Cells	No. of Genes	No. of Cell Types	GEO/EBI Number
Kim et al	Human lung (adenocarcinoma)	208,506	29,634	7	GSE131907
Zilionis et al	Human lung cancers	54,773	41,861	21	GSE127465
Wu et al	Human breast cancers	100,064	29,733	9	GSE176078
Liu	Human breast cancer	118,845	25,301	12	GSE167036
PanglaoDB	Circulating tumor cells in hepatocellular carcinoma	6,475	26,096	9	SRS2737286
PanglaoDB(2)	Circulating tumor cells in hepatocellular carcinoma	3,382	25,790	8	SRS2737287

S6 Benchmarking on Cancer-specific Single-cell RNA-seq Datasets

We benchmarked our proposed method, `scSorterDL`, against nine widely used cell type annotation tools using six publicly available cancer-related single-cell RNA-seq datasets. These datasets span various tumor types including lung and breast cancers, as well as circulating tumor cells from hepatocellular carcinoma. Table S4 summarizes the accuracy across all methods and datasets. "NA" indicates failure due to limitations such as memory exhaustion, insufficient marker coverage, or incompatibility with certain data modalities.

A summary of the cancer datasets used for benchmarking is provided in Table S5. These datasets were collected from human tumor samples and include scRNA-seq profiles from lung adenocarcinoma, breast cancer, and circulating tumor cells. Each dataset varies in the number of cells, gene coverage, and annotated cell types. We also list the GEO or EBI accession numbers for reproducibility and reference.
